# Music Identification System Using MPEG-7 Audio Signature Descriptors

**DOI:** 10.1155/2013/752464

**Published:** 2013-03-07

**Authors:** Shingchern D. You, Wei-Hwa Chen, Woei-Kae Chen

**Affiliations:** ^1^Department of Computer Science and Information Engineering, National Taipei University of Technology, Taipei 104, Taiwan; ^2^Hon-Hai Precision Industry Co. Ltd, Tucheng District, New Taipei City 236, Taiwan

## Abstract

This paper describes a multiresolution system based on MPEG-7 audio signature descriptors for music identification. Such an identification system may be used to detect illegally copied music circulated over the Internet. In the proposed system, low-resolution descriptors are used to search likely candidates, and then full-resolution descriptors are used to identify the unknown (query) audio. With this arrangement, the proposed system achieves both high speed and high accuracy. To deal with the problem that a piece of query audio may not be inside the system's database, we suggest two different methods to find the decision threshold. Simulation results show that the proposed method II can achieve an accuracy of 99.4% for query inputs both inside and outside the database. Overall, it is highly possible to use the proposed system for copyright control.

## 1. Introduction

With the SOPA (stop online piracy act) bill [1] proposed in 2011, the protection of copyrighted intellectual property, such as digital content, once again brought to public attention. Despite the controversial issues of the SOPA bill, it is commonly agreed that copyrighted digital content should be protected. However, the first step toward the protection of copyrighted content is to identify whether a piece of digital content is copyrighted, and if so, who owns it. In this regard, it is important to identify (detect) whether a digital work is copyrighted or not.

Among digital content, soundtracks (usually in the form of audio files) are one type of content that is easily to be illegally reproduced. Owing to the advanced techniques in audio compression, music soundtracks are usually distributed over the Internet in compressed form rather than in uncompressed form. Therefore, any approach for copyright detection must be able to deal with both compressed and uncompressed audio files.

A typical method to attach the copyright information to a piece of music is by embedding watermarks [2]. Though effective, this method has some limitations, such as the watermarks must be embedded into the source soundtracks before release. Therefore, it is not possible to identify the rights owner of a piece of music without watermarks. Another concern is that the embedding process usually introduces distortion. Thus, the quality of the embedded audio may be degraded.

In addition to the watermarking technique, it is also possible to identify rights owner by comparison. For example, if an unknown soundtrack is very similar to a soundtrack owned by a company, then the unknown soundtrack is highly likely copyrighted in that company's name. This type of approach is especially suitable for audio files because, in practice, tremendous amount of records currently available do not embed watermarks or any kind of copyright information.

When comparing a piece of music with a music database, the comparison may be accomplished based on the melody (i.e., musical notes) of the music [3]. For this type of comparison, however, if two persons sing the same song, these two works will be recognized as the same one. Since different artists may perform the same song, known as the cover version, a comparison based on melody cannot solve this problem.

Another type of comparison is based on the waveform of the music. This technique is also known as music identification. In this case, the same song performed by different artists generally does not have the same waveforms, and therefore they can be correctly identified. Though conceptually simple, it is not plausible to directly compare PCM samples of two pieces of music because it would take too much time for the comparison. For example, a typical compact disc (CD) has about 600 M bytes of PCM samples to store about ten songs. If a database contains 10,000 different songs, then the PCM samples occupy about 600 G bytes of space. A piece of unknown music with duration of ten seconds has about 880 k bytes of data. It is obvious that it requires a huge amount of computation to sequentially compare the 880 k bytes of data with the 600 G bytes of data in the database. Therefore, dimension-reduced representations of the PCM waveforms, known as fingerprints, are used in comparison. Among the fingerprints, most of them are defined by individual companies or groups. Some of them are briefly explained in the following.

Researchers in Google develop a fingerprinting scheme for audio called Waveprint [4] based on wavelets. With the aid of wavelets, the fingerprint is invariant to timescale change. In other words, whether the audio piece is played faster or slower than the normal speed, the fingerprint is unchanged. The fingerprint of a piece of 4-minute music is around 64 k bytes, equivalently 2133 bits per second.

Shazam [5] is a company (and service) dedicated for music identification. Its database contains around eleven million soundtracks. As described in [6], the fingerprints used are sets of triplets based on spectrogram peaks. For example, if (*t*_1_, *f*_1_) and (*t*_2_, *f*_2_) are two peaks at time *t*_1_ and *t*_2_ and frequency *f*_1_ and *f*_2_, then the triplet ((*t*_2_ −  *t*_1_), *f*_1_, (*f*_2_ − *f*_1_)) is a feature. Based on the realization of [7], the fingerprint in this scheme uses 400 bits per second.

Researchers in Philips also propose a fingerprinting scheme [8]. The computation of the fingerprints includes: framing, windowing (von Hann window), FFT (fast fourier transform), band decision, energy computation, and then quantization (into binary). In the typical setting, one second of audio has around 2,730 bits of fingerprint.

Microsoft's Robust Audio Recognition Engine (RARE) [9] divides the incoming audio into overlapping frames. Each frame is converted to spectral domain by MCLT (modulated complex lapped transform). The spectral values are applied to two layers of OPCA (oriented principle component analysis) to reduce the dimensionality of the spectral data. For this method, 344 features (11,008 bits if one feature is stored in 4 bytes in a floating point) are obtained per second.

In addition to the above methods, there are actually many more different types of audio fingerprinting schemes available, such as Music Brainz [10], Audible Magic [11], and Gracenote's MusicID [12]. According to [13], there are more than ten different audio fingerprinting schemes available.

Since there are vast amount of different fingerprinting schemes available, some researchers then conducted experiments to compare the relative performance among some of them. The results show that, if the schemes use the same number of bits to represent fingerprints, they have comparable performance [14]. Therefore, the selection of the fingerprinting schemes should also consider other factors (such as interoperability to be addressed below) rather than merely the minor performance difference.

With the ever-increasing amount of multimedia content over the Internet and in the multimedia databases, it is an important task to exchange multimedia content. To respond the public demands, ISO's (International Standardization Organization) working group developed MPEG-7 standard [15, 16]. In the audio part of the standard [17], a high-level tool is developed for audio identification called audio signature description scheme. The fingerprints used in the scheme are called audio signature descriptors, and they have good identification accuracy [18, 19]. In the following, we interchangeably use descriptors and fingerprints without distinction.

Although proprietary audio fingerprints have excellent identification performance, the MPEG-7 audio descriptors offer some advantages. First, being an international standard ensures the open and fair use (subject to license fee) of the technology. Second, such an international standard makes the interoperability possible. For example, if a mobile phone installs an application program to convert a piece of recorded audio to MPEG-7 descriptors, the descriptors can be sent to any website accepting the descriptors. On the other hand, it is not possible to send proprietary fingerprints used in one company to database systems owned by competitors. Third, different companies may share or exchange their audio descriptors (fingerprints) in their databases without any difficulties. In the current situation, each company has to compute fingerprints for newly released albums. With the use of MPEG-7 descriptors, the redundant efforts of computing fingerprints can be minimized.

Although a music identification system based on audio fingerprints has several applications [8], we concentrate on the issue of detecting if a piece of circulated music is highly similar to a copyrighted work or not. In a typical case, the similarity is measured by a distance metric. If the distance is shorter than a threshold, the two pieces of music are considered as similar. Although it is not trivial to determine a suitable threshold [20], this problem is not fully studied. For example, [20] does not indicate any approach to determine the threshold. In addition, the audio files to be compared may be very large; therefore it is very important to reduce the comparison time while maintaining high identification accuracy. Since not many papers address these two issues based on MPEG-7 descriptors, we report in this paper our approaches and experimental results.

This paper is organized as follows. Section 2 is an overview of the MPEG-7 audio signature descriptors. Section 3 is the system model for music identification. Section 4 describes the dimensionality reduction method used in the paper. Section 5 is the proposed strategy to determine the threshold. Section 6 covers the experiments and results. Section 7 is the conclusion.

## 2. Overview of MPEG-7 Audio Signature Descriptors

Part 4 [17] of the MPEG-7 standard includes low-level and high-level descriptors for various applications. Low-level descriptors are derived from the temporal and spectral characteristics of the waveform, whereas the high-level descriptors are constructed based on low-level descriptors. In this section, we will briefly describe the descriptors related to music identification. 

### 2.1. Low-Level and High-Level Descriptors of the MPEG-7 Audio

There are 17 low-level audio descriptors defined in the standard. All of these low-level descriptors are derived from the waveform of the music. They can be divided into six different categories: basic, basic spectral, signal parameter, temporal timbral, spectral timbral, and spectral basis representation. These low-level features may be directly used or may serve as the basics for constructing high-level descriptors.

Based on low-level descriptors, MPEG-7 audio standard also defines high-level description schemes for various applications. These include audio signature description, instrument timbre description, general sound recognition and indexing description, and spoken content description. The audio signature descriptors, one type of fingerprints, are used for music identification.

### 2.2. MPEG-7 Audio Signature Descriptors

Although low-level descriptors in MPEG-7 audio may also be used to identify music, it is shown that the audio signature descriptors provide better identification performance [18, 19]. Therefore, the audio signature descriptors are adopted in the proposed system.

The MPEG-7 audio signature descriptors are computed as follows.

(1) Time-to-frequency conversion: this step is based on audio spectrum envelope descriptors, including the following substeps.
  (i) Determine the hop length between two consecutive windows in the unit of samples. The default value is equivalent to 30 ms.
  (ii) Define the window length *l*_*w*_. This value is set to three times the hop length. The chosen window is Hamming window.
  (iii) Determine the length of FFT, denoted as *N*_FFT_. To reduce the computational complexity, *N*_FFT_ is the smallest power-of-2 number equal to or greater than *l*_*w*_. For example, if the sample rate of the audio is 44,100 s/s, then *l*_*w*_ = 44100 · 0.03 · 3 = 3969. Therefore, *N*_FFT_ = 4096. The extra samples after *l*_*w*_ are padded with zeros.
  (iv) Perform the FFT on the windowed samples.
(2) Divide the FFT coefficients into subbands, with each subband having a bandwidth of one-fourth of an octave. In addition, the bandwidths of two consecutive subbands should overlap each other by 10%. That is, the computed bandwidth for each subband must be multiplied by 1.1. The beginning frequency of the first subband, denoted as loEdge, is fixed at 250 Hz. Table 1 lists the frequency range of the first three subbands before and after (spectral) overlapping.
(3) Find the flatness measure *F*(*b*) for subband *b *by
  (1) $$\begin{array}{c} F\left(b\right)=\frac{\sqrt[{h\left(b\right)-l\left(b\right)+1}]{\prod_{i=l\left(b\right)}^{h(b)}c\left(i\right)}}{\left(1/\left(h\left(b\right)-l\left(b\right)+1\right)\right)\sum_{i=l\left(b\right)}^{h(b)}c\left(i\right)}, \end{array}$$
  where *c*(*i*) is the power spectrum computed by FFT (in step 1), and *h*(*b*) and *l*(*b*) are the lower and upper indices of *c*(*i*) within subband *b*.
(4) Find the mean and variance of *F(b)* for subband b over a certain number of successive FFT windows, called scaling ratio. The default value of the scaling ratio is 16.
(5) The series of mean and variance values are the audio signature descriptors.

**Figure 1:**
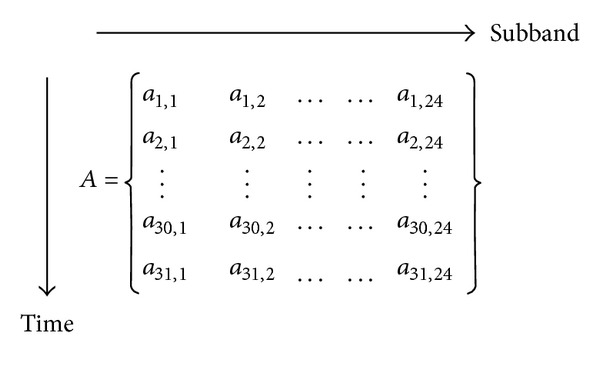
MPEG-7 audio signature descriptors in a matrix.

With the computational steps of the audio signature descriptor, we may compute the number of descriptors in a piece of 15-second music. In the time domain, about (15−0.09)/0.03 = 497 windows are used to cover the 15-second signal because the hop size is 30 ms. Since the scaling ratio is set to 16, there are 497/16 ≈ 31 values per subband. In the spectral domain, since there are four subbands per octave and we use six octaves (250 Hz ~ 16 kHz), there are totally 24 subbands in the spectral domain. Thus, the 15-second signal produces 24 × 31 = 744 mean values and other 744 variance values. The descriptors are arranged in a matrix form, as shown in Figure 1.

**Figure 2:**
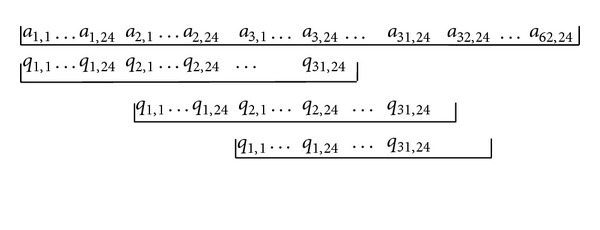
The sliding operation to compare the query input *Q* (15 seconds) and the reference music *A* (30 seconds).

Since the mean values are sufficient for the identification purpose [21], we will not consider the variance descriptors in the following. The obtained descriptors are referred to as high-resolution (or full-dimensional) descriptors. According to [21], if a piece of query music is to be compared with a reference piece, it should be done with a sliding comparison, as shown in Figure 2. In the figure, one segment of line represents descriptors in a piece of music arranged in one dimensional structure. Using the representation in Figure 1, the query segment is arranged as

(2) $$\begin{array}{c} Q=\left[\begin{array}{ccccccc} q_{1,1} & \cdots & q_{1,24} & \cdots & q_{31,1} & \cdots & q_{31,24} \end{array}\right]. \end{array}$$

Since only descriptors from the same subband are to be compared, the hop size between two query segments in Figure 2 is 24 descriptors, or 0.03 · 16 = 0.48 second. In other words, one descriptor (in a subband) represents 0.48 second of audio samples. The smallest Euclidean distance obtained during the sliding operation is recorded as the distance between the query input and the reference music. For example, if the query *Q* is to be compared with reference *A*, then we may obtain the Euclidean distance *E*_*A*,*B*_(*k*) for the *k*-th sliding comparison as

(3) $$\begin{array}{c} E_{Q,A}\left(k\right)=\sum_{i=1}^{31}\sum_{j=1}^{24}\left|q_{i,j}-a_{(i+k),j}\right|. \end{array}$$

The recorded distance between these two pieces of music is

(4) $$\begin{array}{c} d_{Q,A}=\underset{k}{\min}E_{Q,A}\left(k\right). \end{array}$$

Although using audio signature descriptors greatly reduces the computational burden for comparison, the required computation using (3) is still very large. According to [21], suppose that a database contains 1,000 audio files, with each one having duration of 30 seconds, and the music to be identified has a duration of 15 seconds, then 47, 616, 000 arithmetic operations are required. Therefore, it is beneficial to further reduce the number of comparison, which can be accomplished by employing a multiresolution strategy.

## 3. Music Identification Based on Multiresolution Strategy

As discussed previously, the computational cost is still very high even if we use the MPEG-7 descriptors. Therefore, we will use the multiresolution strategy to reduce the computational complexity. To do so, in addition to the above-mentioned high-resolution descriptors, we also need to generate low-resolution descriptors (see also Section 4). Therefore, the music database contains high-resolution and low-resolution descriptors for each soundtrack to be identified. This step can be accomplished during the setup of the database. In addition, a training process is conducted to find a distance threshold to determine whether the query input is in the database or not. Section 5 has a detailed description about the training process.

**Figure 3:**
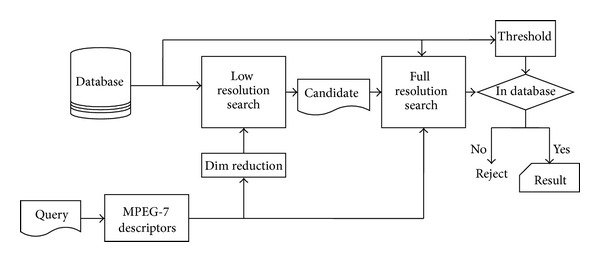
Procedure for multiresolution music identification.

Once the database is constructed, as shown in Figure 3, the music identification procedure consists of the following steps:

1. When a query input is sent to the system, it computes the MPEG-7 audio signature descriptors and low-resolution descriptors for the query music. If a mobile device is used to record the music, usually the descriptors (fingerprints) are sent instead of the PCM samples to reduce the size of the transmitted data.
2. The computed low-resolution descriptors are compared with those in the database. Based on the distance metric, a list of candidates is obtained. Since the query music may start at any position of the soundtrack, a sliding comparison, as shown in Figure 2, is necessary.
3. After obtaining the candidate list, high-resolution descriptors are used to find the distances between the query input and the candidates. The shortest distance and its associated soundtrack are recorded.
4. If the shortest distance is less than the threshold, the query input is considered as highly similar to the recorded soundtrack in the database. Otherwise, the query input is not in the database.

During the low-resolution comparison, it is also possible to use an existing algorithm to reduce the comparison time. For example, we may arrange the descriptors in *k-d* tree (*k*-dimensional binary search tree) [22, 23] structure to reduce the search time. However, using the *k-d* tree structure implies that every segment of music in the database has the same number of descriptors, or same duration. Note that the unit to be compared is one segment, and one soundtrack can be divided into many overlapped segments, as shown in Figure 2. Therefore, if the duration of the segment is set to 15 seconds, then the query music must also have a duration of 15 seconds to use the system. In a practical situation, if the query input is longer than 15 seconds, then only 15 seconds of the music is used for computing the descriptors and comparison.

## 4. Dimensionality Reduction Method for MPEG-7 Audio Signature Descriptors

This section briefly explains the dimensionality reduction technique used in the experiments. Although a block-average method is proposed in [21] for this purpose, we use a different technique in this paper.

### 4.1. Problems of Reducing Dimensionality Using Scaling Ratio

**Figure 4:**
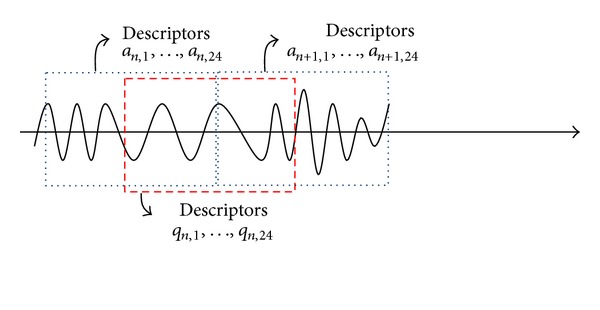
The audio samples and the descriptors. In a real application, *a*_*n*,*k*_ is stored in the database, whereas *q*_*n*,*k*_ is computed from the query input. Usually *a*_*n*,*k*_ is not equal to *q*_*n*,*k*_ due to different scopes of the windows even though they are all derived from the same soundtrack.

Conceptually we may increase the scaling ratio (given in Section 2) to reduce the number of descriptors during computing them. For example, by increasing the scaling ratio from 16 to 256, the number of descriptors is reduced by 16 times. Unfortunately, this approach does not yield satisfactory results because of insufficient time resolution. Recall that the descriptors are derived based on the windowed waveform. Therefore, if two segments of the soundtrack are not highly similar, their corresponding descriptors usually have large differences. With a scaling ratio of 256, each descriptor represents around 7.7 seconds of audio samples. Therefore, unless the query input also starts at a point very close to the segment boundary of a soundtrack, a comparison based on these descriptors may fail. As illustrated in Figure 4, descriptors of *a*_*n*,*k*_ (or *a*_*n*+1,*k*_) and *q*_*n*,*k*_ are quite different, and therefore the descriptor-based identification scheme cannot identify the query input. Therefore, a suitable time resolution, for example, 0.48 second, should be maintained.

### 4.2. Proposed Dimensionality Reduction Method

**Figure 5:**
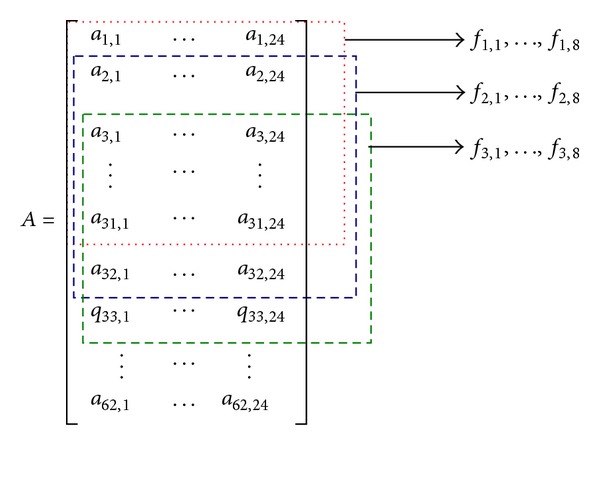
Low-resolution descriptors. In the figure, each temporal-spectral block corresponds to descriptors from 15-second music.

In contrast to reduce the number of descriptors by increasing the scaling ratio, we may reduce them in each temporal-spectral block (i.e., the matrix in Figure 1) representing a segment of (15-second) music. However, to maintain a high time resolution, successive temporal-spectral block should have a small time difference, as shown in Figure 5. With this arrangement, we achieve both high identification rate and low comparison complexity.

**Figure 6:**
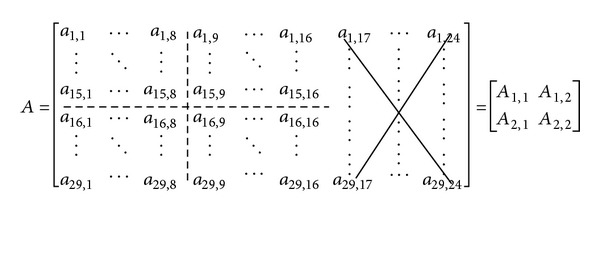
Partition of a temporal-spectral block into four subblocks in the experiments.

For each temporal-spectral block, we use PCA (principal component analysis) [21, 24] to reduce the number of descriptors. Since it is difficult to directly use PCA to obtain good low-resolution descriptors [21], we use an alternative approach. Its basic idea is to partition the entire block into four subblocks, as shown in Figure 6, and then use PCA to reduce the number of descriptors of each subblock into two values (descriptors), called low-resolution descriptors. Also, high-frequency descriptors in the original temporal-spectral block are all discarded because they are susceptible to noise [21]. With this arrangement, totally eight descriptors are used to represent a segment of 15-second music. Note that the actual duration of a segment used in the experiments is 14.04 seconds (though we still say 15 seconds) because some audio samples are lost after MP-3 compression and decompression in the experiments.

We now describe how to calculate low-resolution descriptors. Since using PCA for dimensionality reduction is a well-known approach, we only describe how to construct the covariance matrix for PCA computation and omit the computation of finding principal components. Suppose that there are *N* segments of music pieces in the database, with their descriptor matrices denoted as *A* ^(1)^ to *A* ^(*N*)^. To simplify the argument, we consider the subblock matrix *A*_1,1_^(*k*)^ (referring to Figure 6) in the following. Other subblocks can be computed by the same procedure. First, collect all subblocks from *A*_1,1_^(*k*)^ to form a big matrix as follows:

(5) B=[a1,1(1)⋯a1,8(1)a1,1(2)⋯a1,8(2)⋯a1,1(N)⋯a1,8(N)⋮⋱⋮⋮⋱⋮⋯⋮⋱⋮a15,1(1)⋯a15,8(1)a15,1(2)⋯a15,8(2)⋯a15,1(N)⋯a15,8(N)] =[↑⋯↑b1⋯b8N↓⋯↓].

Then, we may consider column vectors of matrix *B* as **b**_*i*_ vectors. Having the data vectors **b**_*i*_, the covariance matrix and, subsequently, the principal components can be found. By keeping only two principal components, we can obtain **c**_*i*_ = [*c*_*i*,1_ *c*_*i*,2_] from **b**_*i*_. Since there are eight column vectors in a subblock, descriptors in *A*_1,1_^(*k*)^ are reduced to eight **c**_*i*_  (8*k* − 7 ≤ *i* ≤ 8*k*) vectors. Next, we can rearrange the obtained **c**_*i*_ vectors as

(6) $$\begin{array}{c} C=\left[\begin{array}{ccccccc} c_{1,1} & c_{1,2} & c_{9,1} & c_{9,2} & \cdots & c_{8N-7,1} & c_{8N-7,2}\\ \vdots & \vdots & \vdots & \vdots & \cdots & \vdots & \vdots \\ c_{8,1} & c_{8,2} & c_{16,1} & c_{16,2} & \cdots & c_{8N,1} & c_{8N,2} \end{array}\right]. \end{array}$$

Again, by treating each row of matrix *C* as the data to be processed by PCA, we can compute the covariance matrix, and finally reduce each column vector to one value. Since there are two columns originally from one subblock, there are totally two (low-resolution) descriptors per subblock. Equivalently, a segment of 15-second music is represented by eight descriptors. During the computation, the principal components obtained in the first and the second steps should be stored in the database. When a query input (in the form of high-resolution descriptors) is supplied, these components are used to obtain the low-resolution descriptors for the input.

## 5. Threshold to Determine the Membership of the Query Input

Since, in practice, the database cannot collect all music soundtracks in the world, we have to have a strategy to determine if the query input is actually in the database or not. In our case, the decision is accomplished with the aid of a threshold. If the shortest distances between the input and the candidates are greater than a threshold, then the query input is not in the database; otherwise, it is in the database. Though the concept is simple, it is not trivial to determine the threshold [20].

Recall that when a query input is applied to the proposed system, the system computes the Euclidean distances between the low-resolution descriptors of the query input and those in the database. Accordingly, *m *(say, *m* = 20) segments of soundtracks from distinct titles are selected based on the computed distances. Next, the Euclidean distances between the query and the selected segments are computed again using full-resolution descriptors. By sorting the distances from small to large, the system creates a candidate list for the query input.

**Figure 7:**
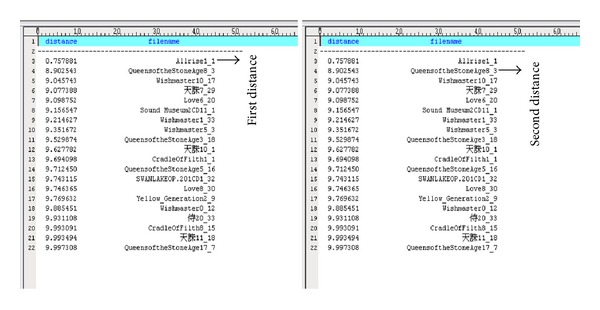
The first and second distances in method I.

To compute the previously mentioned threshold, we examine two different methods. In the first method (method I), we define the “first” distance *d*_1_ as the (full-resolution) distance associated with the first candidate in the list. Similarly, the “second” distance *d*_2_ is the distance associated with the second candidate, as shown in Figure 7. The method to compute the first and second distances is used both in training and in identification. For the training phase, assume that there are *N*_*T*_ soundtracks used. For a segment from a soundtrack indexed *n*, let the first distance be *d*_1_(*n*) and the second distance be *d*_2_(*n*). The threshold *T*_*A*_ is then computed as

(7) $$\begin{array}{c} T_{A}=0.5\cdot \left(\sum_{n=1}^{N_{T}}d_{1}\left(n\right)+\sum_{n=1}^{N_{T}}d_{2}\left(n\right)\right). \end{array}$$

During the identification phase, the first distance for the query input is computed. If the first distance is smaller than the threshold *T*_*A*_, the query input is determined to be highly similar to the top soundtrack title in the candidate list. Otherwise, the query input is not inside the database.

**Figure 8:**
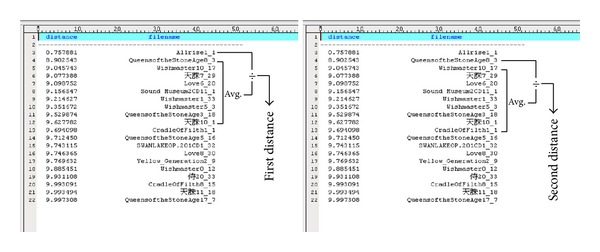
The first and second distances in method II.

In addition to method I, we also propose an alternative method (method II) to compute the first and second distances and the threshold. Referring to Figure 8, the first distance *d*_1_′(*n*) of method II for a training segment in soundtrack *n *is

(8) $$\begin{array}{c} d_{1}^{\prime }\left(n\right)=d_{1}\left(n\right)÷\frac{\sum_{m=2}^{M}d_{m}\left(n\right)}{M-1}, \end{array}$$

where *d*_*m*_(*n*) is the distance associated with the *m*th candidate in the list, and *M* is a constant. Based on our experiment, *M* = 10 is sufficient. By the same arrangement, the second distance *d*_2_′(*n*) is calculated as

(9) $$\begin{array}{c} d_{2}^{\prime }\left(n\right)=d_{2}\left(n\right)÷\frac{\sum_{m=3}^{M+1}d_{m}\left(n\right)}{M-1}. \end{array}$$

Similar to (7), the threshold *T*_*A*_′ is determined as

(10) $$\begin{array}{c} T_{A}^{\prime }=0.5\cdot \left(\sum_{n=1}^{N_{T}}d_{1}^{\prime }\left(n\right)+\sum_{n=1}^{N_{T}}d_{2}^{\prime }\left(n\right)\right). \end{array}$$

During identification phase, if the computed distance *d*_1_′ is greater than*T*_*A*_′, the query input is determined as not in the database. Otherwise, the query input is highly similar to the top soundtrack title in the candidate list.

## 6. Experiments and Results

Before conducting the experiments, we collect 750 soundtracks from many CD titles for constructing the database and for identification. In the experiments, 30 seconds of music is excerpted from the soundtracks as the reference items to be identified. Then, low- and full-resolution descriptors of the reference items are calculated and stored in the database. We also randomly excerpt 15-second query items from the reference items. Note that a query item may start from any sample on the first half of the reference. To test the identification accuracy for compressed audio, the 15-second query inputs are also encoded and then decoded with an MP-3 coder with bitrates of 192 k and 96 k, respectively. However, the database only contains descriptors from the uncompressed items. In addition, the principal components are also obtained using uncompressed items.

Several experiments are conducted to examine the performance of the proposed system. The first experiment checks the identification accuracy using high-resolution descriptors. The second one compares the relative identification accuracy between the proposed method (in Section 4.2) and the method given in [21]. The third experiment compares the search speed by using the linear search and *k-d* tree search. The next experiment intends to determine a suitable value of *M* used in (8) and (9). Having the value of *M*, we report the identification accuracy using a multiresolution strategy in experiment five. In this experiment, both method I and method II are examined. To have a complete comparison between method I and II, we also report the results in terms of the ROC (receiver operating characteristics) [25] curve and the DET-like (detection error tradeoff) [26] curve but without logarithm.

### 6.1. Experiment One: Identification Accuracy Using High-Resolution Descriptors

The first experiment is to examine the identification accuracy using the high-resolution MPEG-7 audio signature descriptors (without reduction). To evaluate the influences of high-frequency descriptors, we also examine the accuracies with and without using high-frequency (greater than 2.5 kHz) descriptors. The results are given in Table 2. In the table, “match in first one” is the rate that the query input is correctly identified with the shortest distance among all reference items. Similarly, “match in first 15” is the rate that the query is correctly identified within a list of 15 reference items sorted by distance. The results indicate that high-resolution descriptors have very good identification accuracy. Also, for uncompressed or 192 k MP-3 items, whether using high-frequency descriptors does not affect the identification accuracy. However, for 96 k MP-3 items, discarding high-frequency descriptors greatly improves the accuracy.

### 6.2. Experiment Two: Identification Accuracy Using Low-Resolution Descriptors

The second experiment compares the relative identification accuracies of the proposed dimensionality reduction technique and the averaging method proposed in [21]. In this experiment, eight descriptors are used to represent one segment of music (15 seconds). The results are shown in Table 3. For this experiment, the accuracy rate in “match in first 15” is more important than the figure in “match in first one” because the low-resolution descriptors are used to produce a candidate list. Therefore, it is acceptable as long as the correct item is within the list, as the high-resolution descriptors are to be used in the second stage. In this regard, the proposed approach is slightly better than the averaging method proposed in [21] for 96 k MP-3 query inputs.

### 6.3. Experiment Three: Time for *k-d* Tree Search and Linear Search

The third experiment compares the time to generate a list of 20 candidates by using *k-d* tree search and linear (exhausted) search for low-resolution descriptors. The computing time is obtained by averaging the required time in ten trials. For each trial, 500 query items are randomly selected among the 750 available items. The experimental platform is a personal computer with a 1.6 GHz Pentium CPU and 1.5 GB memory. The program is written in C with a Borland C++ compiler. The results are shown in Table 4. The results show that we can increase the search time by 10-fold if the low-resolution descriptors are embedded in the *k-d* tree structure.

### 6.4. Experiment Four: Determination of *M* in Method II

**Figure 9:**
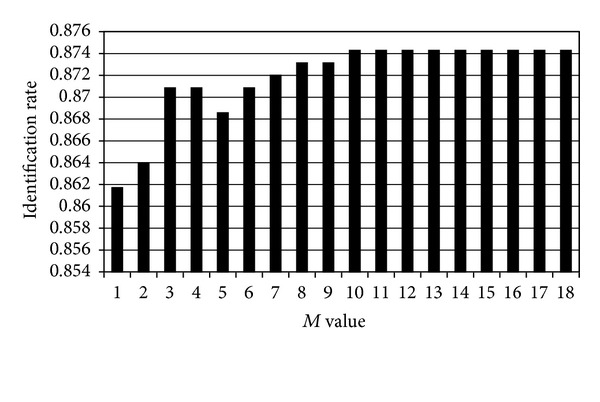
The identification rate versus the number *M* for 96 k MP-3 query inputs.

The fourth experiment is to determine the number *M* in method II. Recall that the thresholds in both methods are computed based on high-resolution descriptors. To determine the optimal value of *M*, we vary this value from one to eighteen in (8) and (9) to compute the threshold *T*_*A*_′ and then to perform identification. Note that method II is degenerated to method I if *M* = 1. In this experiment, 96 k MP-3-coded items are used as the query. In addition, we deliberately keep the high-frequency descriptors in computing the distances because keeping them makes it easier to examine the influence of *M* versus identification rate (defined in experiment five). The experimental results are given in Figure 9. The results show that when *M* is equal or greater than 10, the identification rate remains almost constant. Therefore, *M* = 10 is sufficient for method II. This value is then used in experiment five.

### 6.5. Experiment Five: Comparison between Method I and Method II

The fifth experiment compares the identification rates between method I and method II. The thresholds are calculated using (7) and (10), respectively. In this experiment, 375 of the 750 15-second items are randomly chosen for training (i.e., to obtain the thresholds). The rest of 375 items are for query. Note that all of the items are excerpted from the reference items in the database. In addition, we test other 500 query items (also with duration of 15 sec) not excerpted from database references.

Since some query inputs are not in the database, we also need to consider the situations of falsely identifying an outside database item as one inside the database, and vice versa. Suppose that *T*_*d*_ query items are actually inside the database and *T*_*o*_ query items are not inside the database. Assume that the identification system correctly identifies *N*_*d*_ inside-database query item, erroneously rejects *N*_*r*_ inside-database query items, and erroneously accepts *N*_*a*_ outside database query items. Then, the IDR (identification rate), FAR (false-accept rate), and FRR (false-rejection rate) are computed as follows:

(11) IDR=NdTd−Nr

(12) FAR=NaTo

(13) FRR=NrTd.

In addition, the accuracy of the system is computed as

(14) $$\begin{array}{c} \text{ACC}=\frac{N_{d}+N_{o}}{T_{d}+T_{o}}, \end{array}$$

where *N*_*o*_ is the number of query items correctly identified as outside-database items and can be computed as *N*_*o*_ = *T*_*o*_ − *N*_*a*_.

In this experiment, the low-resolution descriptors are used to find 20 candidates. Next, high-resolution descriptors are used to find the best-matched one in the list. If the distance associated with the best-match is greater than the threshold, the query input is determined as not in the database. Otherwise, the query input is identified as the best-matched one. In this experiment, high-frequency descriptors are kept in the comparison. With the use of (11) to (14), the average values after four trials are given in Table 5. From the results we know that the system does not perform well for identifying 96 k MP-3 query items. This is mainly due to high distortion in high-frequency descriptors. A similar phenomenon can also be observed in Table 2. Later on, we will repeat this experiment but discard high-frequency descriptors.

**Figure 10:**
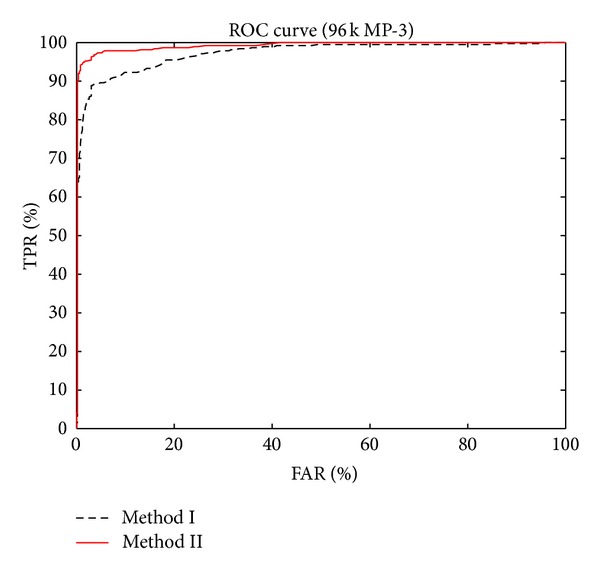
The ROC curves for both methods.

**Figure 11:**
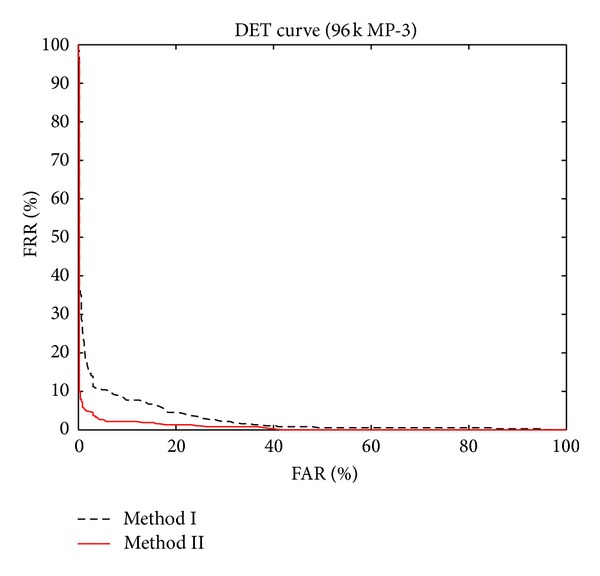
The DET-like curves without logarithm for both methods.

To further examine the performance differences between method I and method II, we again use 96 k MP-3 items as the query inputs. But this time we vary the threshold values (for both methods) and then record the IDR, FAR, and FRR values. The results are presented as ROC curves [25] and DET-like curves [26] (but without logarithm), as shown in Figures 10 and 11, respectively. The ROC curve plots FAR versus TPR (true positive rate), which is defined as

(15) $$\begin{array}{c} \text{TPR}=\frac{N_{d}}{T_{d}}. \end{array}$$

Conceptually, a better classifier should have a higher TPR for a fixed FAR. Therefore, a better classifier should have an ROC curve closer to the left-upper corner. With this interpretation, from Figure 10 we know that method II is a better classifier.

In this experiment, we also show DET-like curves. A “true” DET curve plots FAR versus FRR using logarithm scales. In our case, however, we do not use logarithm scales to exhibit the similarity between ROC and DET curves. Again, conceptually we know that a better classifier should have a lower FRR over a fixed FAR. Thus, a better classifier should have a curve closer to the left-bottom corner. By examining Figure 11, we again confirm that method II is a better method.

Since the high-frequency descriptors actually affect the identification rate at low bitrates, we again repeat this experiment without using high-frequency descriptors. The results are given in Table 6. By comparing the results in Tables 5 and 6, we know that removing high-frequency descriptors slightly reduces the accuracy of uncompressed items. However, it greatly improves the accuracy for 96 k MP-3 items. Since method II has an accuracy of at least 99.4% in all cases, it is highly plausible to identify copyrighted audio materials using this method.

## 7. Conclusion

In this paper, we propose a system using a multiresolution strategy to identify whether a piece of unknown music is identical to one of the pieces in the database. In the system, high-resolution descriptors are MPEG-7 audio signature descriptors, and the low-resolution descriptors are obtained from high-resolution descriptors with the aid of PCA to reduce their dimensionality. Experimental results show that low-resolution descriptors still have high identification accuracies. To reduce the time to generate the candidate list, we use the *k-d* tree structure to store low-resolution descriptors. Experimental results show that using *k-d* tree structure increases the search time by ten-folds. Since not every piece of query input is within the database, we also proposed two methods to determine the distance thresholds. Experimental results show that the proposed method II provides an accuracy of 99.4%. Therefore, the proposed system can be used in real applications, such as identifying copyrighted audio files circulated over the Internet. As it can be easily extended to operate with multiple computers, the proposed system is a plausible starting point to construct a large, operable database.

## Figures and Tables

**Table 1:** Bandwidth of the first three subbands.

Bandwidth of subband (nonoverlapped)	Bandwidth of subband (overlapped)
250–297.3 Hz	237.5–312.2 Hz
297.3–353.6 Hz	282.4–371.2 Hz
353.5–420.4 Hz	335.9–441.5 Hz

**Table 2:** Identification accuracy using high-resolution features.

	Uncompressed		192 k MP-3		96 k MP-3	
High-frequency descriptors	Used	Not used	Used	Not used	Used	Not used
Match in first one	100%	100%	100%	100%	94%	100%
Match in first 15	100%	100%	100%	100%	98%	100%

**Table 3:** Comparison of identification accuracies using low-resolution descriptors obtained by the proposed approach and by the averaging (avg) method [21].

	Uncompressed		192 k MP-3		96 k MP-3	
Method	Proposed	Avg.	Proposed	Avg.	Proposed	Avg.
Match in first one	99.3%	99.5%	99.1%	99.3%	90.1%	89.8%
Match in first 15	100%	100%	100%	100%	99.6%	99.5%

**Table 4:** Comparison of search time between *k*-*d* tree search and linear search.

	*K*-*d* tree	Linear search
Average time	0.255 sec	3.23 sec

**Table 5:** Comparison of identification performance using method I and method II with the use of high-frequency descriptors.

	Uncompressed		192 k MP-3		96 k MP-3	
	Method I	Method II	Method I	Method II	Method I	Method II
IDR	98.8%	99.9%	99%	99.7%	84.1%	87.4%
FAR	2.1%	0.2%	1.8%	0.2%	0.4%	0.2%
FRR	0.0%	0.0%	0.0%	0.5%	36.6%	29.6%
ACC	98.4%	99.9%	98.9%	99.5%	71.8%	76.8%

**Table 6:** Comparison of method I and method II for IDR, FAR, and FRR with high-frequency descriptors removed.

	Uncompressed		192 k MP-3		96 k MP-3	
	Method I	Method II	Method I	Method II	Method I	Method II
IDR	99.1%	99.7%	98.9%	99.7%	99.0%	99.7%
FAR	1.5%	0.2%	1.9%	0.2%	1.6%	0.2%
FRR	0.3%	0.4%	0.2%	0.4%	0.3%	0.6%
ACC	98.7%	99.5%	98.5%	99.5%	98.6%	99.4%
